# Supplementary material for: VesiclePy: A machine learning vesicle analysis toolbox for volume electron microscopy
Source: PLoS Comput Biol. 2026 May 14;22(5):e1013499. doi: 10.1371/journal.pcbi.1013499 (PMC13211309; doi:10.1371/journal.pcbi.1013499)
Supplement: Attachment — Submitted filename: revision_letter.pdf [file pcbi.1013499.s001.pdf]

Response to Reviewers

We thank the reviewers for their careful reading of our manuscript and their constructive comments. Their feedback has helped improve the clarity, completeness, and rigor of the manuscript. Below we provide a point-by-point response to each comment and describe the revisions made in the manuscript.

For each response, we indicate where the corresponding changes were made in the revised manuscript and provide excerpts where appropriate.

Additionally, we have addressed technical issues from the initial submission to meet PLOS Computational Biology's Software article guidelines.

Reviewer #1

Comment 1

> The manuscript lacks explicit, self-contained descriptions of the dataset. Relying on prior work without providing related details may confuse readers about the data used here. Please describe key dataset information directly in the manuscript, including resolution and imaging modality. For example, in the cited prior work [5], the sample was "Approximately half of the sample (0.05 mm thickness) was then cut along the oral-aboral plane into 1,829 thin sections (30 nm thick), collected and imaged with a scanning electron microscope at 4 nm resolution, and stitched and aligned to form image stacks." Please describe these specifics (resolution, sectioning, imaging modality, alignment/stitched stacks) within the present study.

Response

We thank the reviewer for pointing out that the dataset description should be self-contained. In the revised manuscript, we added a detailed description of the electron microscopy dataset, including section thickness, voxel resolution, imaging modality, acquisition parameters, and alignment procedures. This ensures that the dataset used in this study is fully described without requiring readers to consult the cited work.

Where addressed in manuscript

Section: **Results**

Lines: **268-275**

Revised text:

> “The example dataset consists of a scanning electron microscopy (SEM) volume acquired from high-pressure frozen Hydra vulgaris. Serial sections were collected at 30 nm thickness, yielding an effective voxel resolution of 4 × 4 × 30 nm. Imaging was performed on a Zeiss SIGMA scanning electron microscope using Atlas 5 (Zeiss Microscopy) mapping software, with acquisition parameters of 1000 ns dwell time, 4 nm in-plane pixel size, and back-scattered electron detection. The resulting EM sections were subsequently stitched and aligned using the open-source Python package feabas [30].”

Comment 2

> The method has been evaluated only on Hydra vulgaris datasets and has not been extended to other datasets. Clarify whether the method is applicable to other vEM datasets (e.g., FlyWire, MICrONS, H01) or to other vesicle types, or whether it is limited to Hydra vulgaris. Also address whether this workflow represents the first fully end-to-end analysis toolbox in the field and articulate the main innovations. The discussion, limitations, and contributions could be strengthened with explicit statements about generalizability and scope, or with preliminary results on additional datasets if feasible.

Response

We appreciate the reviewer’s suggestion to clarify the scope and generalizability of the method. In the revised manuscript, we expanded the description of the pipeline design to emphasize that VesiclePy is not organism-specific and can be applied to other volume electron microscopy datasets. We added discussion of domain shift arising from differences in fixation protocol, imaging modality, voxel anisotropy, and signal-to-noise characteristics, and we describe how the segmentation model can be retrained or fine-tuned using additional ground-truth annotations. We also strengthened the description of the pipeline’s novelty as a unified end-to-end framework integrating vesicle segmentation, classification, spatial analysis, and neuron-level clustering.

Where addressed in manuscript

Section: **Design and Implementation**

Lines: **41-53**

Revised text:

> "Although demonstrated here on high-pressure frozen Hydra vulgaris serial SEM data, the modular design of VesiclePy does not rely on organism-specific priors. The segmentation backbone (3D U-Net with watershed decoding) operates on voxel-level contrast and spatial continuity and can be retrained on other vEM datasets given appropriate ground-truth annotations. In principle, the pipeline can be applied to other large-scale volume electron microscopy datasets, including connectomics resources such as FlyWire [6], MICrONS [7], or the H01 datasets [8], provided that appropriate vesicle annotations are available for model training or fine-tuning.
>
> Nevertheless, performance may be affected by domain shift arising from differences in fixation protocol, imaging modality, voxel anisotropy, or signal-to-noise characteristics. In such cases, fine-tuning using a small curated subset of labeled volumes is recommended. The iterative human-in-the-loop correction cycle implemented in VesiclePy is designed to mitigate such cross-dataset variability efficiently."

Comment 3

> The claim that "VesiclePy can process a multiterabyte serial EM dataset" is compelling, but the manuscript does not provide concrete demonstrations of data size, throughput, or practical resource/time requirements. Please include: dataset sizes used in experiments, processing times, hardware specifications (CPU/GPU, RAM, storage), and any parallelization strategies. A discussion of bottlenecks and scalability would be helpful.

Response

We thank the reviewer for suggesting that the scalability claims be supported with concrete metrics. In the revised manuscript we added details describing dataset size, computational infrastructure, and runtime requirements. Specifically, we now report the size of the original EM volume and the analyzed neuron volumes, the total number of vesicles processed, the hardware specifications used for computation, and the parallelization strategy used for large-scale processing. We also describe the use of Slurm-based workload distribution and chunked HDF5 storage to enable efficient out-of-core processing.

Where addressed in manuscript

Section: **Results**

Lines: **288-304**

Revised text:

> “Dataset-wide processing was executed on a high-performance computing cluster utilizing two dedicated compute nodes, each equipped with 64 CPU cores, 512 GB of RAM, and four NVIDIA A10 GPUs (24GB VRAM each). The most computationally intensive component of the VesiclePy workflow is the training of the 3D U-Net segmentation model. In our experiments, model training required approximately 10 hours using four NVIDIA A10 GPUs.
>
> Once the segmentation model has been trained, the remaining stages of the pipeline—including vesicle classification, spatial analysis, and visualization—are substantially lighter computationally. In particular, many downstream analyses operate on compact point cloud representations of vesicles rather than the original volumetric data, enabling efficient processing of tens of thousands of vesicles with minimal memory overhead.
>
> While the full aligned serial EM volume spans approximately 45.9 trillion voxels (~45.9 TB at uint8), our analysis focused on the cumulative image data contained within the 20 individual neuronal bounding boxes, which totaled 2.75 TB. To facilitate this scale of analysis, workload distribution was managed via Slurm-based job sharding, utilizing a chunked HDF5 storage backend with Gzip compression to maintain an out-of-core parallelization strategy.”

Comment 4

> For the 3D U-Net outputs (distance transform and edge maps), clarify whether these are computed in 2D or 3D, and discuss how the choice impacts seed generation. Also elaborate on the statement “we collapsed the three-channel prediction into a single-channel prediction of individual vesicles.” Provide sufficient detail to explain how the reduction is performed and how potential contradictions between channels are mitigated. It would help to illustrate potential failure cases, such as the yellow vesicle with holes in Fig. 3B and the partially reconstructed green vesicles in Fig. 3D, and discuss how the method addresses such issues.

Response

We appreciate the reviewer’s request for clarification. In the revised manuscript we expanded the description of the instance segmentation pipeline to explicitly describe how the three prediction channels are generated and used during seeded watershed segmentation. We also clarified how contradictions between prediction channels are handled and expanded the discussion of failure cases illustrated in Fig. 3. These revisions provide a more transparent description of the instance segmentation workflow.

Where addressed in manuscript

Section: **Large Vesicle Instance Segmentation: Instance segmentation**

Lines: **97-116**

Revised text:

> “To achieve precise instance segmentation of vesicles, we predicted a three-channel volume comprising binary segmentation, contour, and distance transform maps. While the 3D U-Net processes volumetric context and outputs the distance transform as a 3D target, the contour map is generated slice-wise (2D) to capture precise in-plane boundaries.
>
> We collapsed this multi-channel prediction into single-channel labeled instances using a 3D seeded watershed algorithm. Crucially, seeds are not generated via simple local maxima detection; instead, they are generated via a strict threshold intersection across all three channels (high binary, low contour, high distance). Following connected-components labeling and small-seed removal, these regions serve as the starting points for flooding.
>
> To mitigate contradictions between channels—such as the contour map predicting a boundary where the binary mask predicts foreground—the algorithm applies a strict hierarchy. The contour channel acts primarily as a seed suppressor, while the foreground mask is defined by the binary and distance channels. Failure cases, such as vesicles with holes (Fig. 3B) or partial reconstructions (Fig. 3D), typically arise from noisy binary predictions and are addressed through iterative sampling of erroneous subvolumes during subsequent training cycles.”

Comment 5

> In the Unsupervised Small Vesicle Type Classification section, vesicles defined as diameter < 80 nm may span 2–3 z-slices, yet the self-supervised classification uses 2D patches (11×11). Clarify whether edge-position patches could affect classification, and whether extending the receptive field in the z-dimension or using sequential 3D patches could improve results. Regarding the variational autoencoder features, what is the dimensionality of the latent embedding? The manuscript states “Each 11×11 image patch is compressed into an embedding with two latent dimensions.” Specify the exact technique (e.g., PCA or a VAE encoder) and rationale. For Fig. 5, indicate what the x- and y-axes represent (e.g., PCA projections) and label them accordingly if PCA is used (PCA Projection 1, PCA Projection 2).

Response

We thank the reviewer for requesting clarification of the unsupervised classification procedure. In the revised manuscript we clarified how the 2D patch is extracted from the geometric center of each vesicle's 3D bounding box, which minimizes edge-slice artifacts. We also clarified that the embeddings are generated by a convolutional variational autoencoder with a two-dimensional latent space and that the visualization axes correspond to the latent variables produced by the encoder.

Where addressed in manuscript

Section: **Unsupervised Small Vesicle Type Classification**

Lines: **159-172**

Revised text:

> "Although small vesicles may span 2–3 z-slices, the pipeline explicitly calculates the 3D bounding box midpoint for each vesicle and extracts exactly one 2D slice at this geometric center. No maximum intensity projection (MIP) is used. This center-slice extraction inherently avoids edge-position slices (the top or bottom caps of the vesicle), which typically suffer from partial-volume effects and lower contrast.
>
> The VAE latent dimensionality is fixed at 2. Consequently, each 11 × 11 image patch is compressed into a two-dimensional embedding, where the plotted x- and y-axes represent the raw VAE latent means (μ_1 and μ_2) generated by the encoder; no Principal Component Analysis (PCA) or further dimensionality reduction is applied."

Comment 6

> Several figures would benefit from additional annotations. For Fig. 4, include a scale bar and clarify what the red contour lines represent, especially if they do not perfectly align with vesicle boundaries. For Fig. 8, define the vertical axis precisely. Ensure all captions and overlays consistently explain color codes, contours, and annotations to avoid confusion.

Response

We thank the reviewer for this suggestion. In the revised manuscript we clarified the figure captions and annotations. The caption of Fig. 4 now explicitly explains that the red contours represent predicted vesicle boundaries from the segmentation model. We also clarified the meaning of the vertical axis in Fig. 8, specifying that it represents the Gower dissimilarity used in hierarchical clustering and have labeled the figure accordingly.

Where addressed in manuscript

Fig. 4 caption

> "The interface enables human-in-the-loop verification of automated vesicle classifications. For each candidate (A) Clear vesicle (CV), (B) Dense core vesicle (DCV), or (C) Dense core vesicle with a halo (DCVH), the interface presents five consecutive Z-slices (Slices 0–4) overlaid with the contour of the 3D U-Net prediction in red, allowing users to rapidly assess volumetric morphology and label accuracy. Scale bar: 200 nm."

Section: **Neuron Type Cluster Analysis**

Lines: **258-262**

Revised text:

> "The vertical axis of the resulting dendrogram represents the absolute Gower dissimilarity, which calculates the average difference across the morphological and organellar features. In this representation, a height of 0.0 signifies identical feature profiles, while 1.0 represents the maximal theoretical divergence across all parameters."

Comment 7

> Clarify potential citation numbering (e.g., "NucMM challenge (19)" may refer to [19]), and ensure figure references follow journal conventions (e.g., "Fig. x" rather than "Fig x"). A careful language and formatting check is recommended.

Response

We performed a comprehensive language and formatting review of the manuscript. Figure references have been standardized to follow the journal's "Fig." convention, and citation formatting has been verified throughout the manuscript.

Reviewer #2

Comment 1

> Some expressions in the manuscript may be improved. For example, from line 28–30: "Segmentation consists of an iterative deep learning model to automatically

> segment large vesicles from ground truth data, and manual segmentation of small vesicles." I guess a more direct expression may be like this: "A deep learning model which is iteratively trained with ground-truth data is used to segment large vesicles, and for difficult segmentation of small vesicles, a manual segmentation tool is provided."

Response

We thank the reviewer for this suggestion. The sentence has been revised to improve clarity and better reflect the workflow of the segmentation pipeline. Additionally, we have revised several other instances of roundabout or otherwise awkward phrasing throughout the manuscript.

Comment 2

> Colormap representations in figure 5 and 6 should be clarified.

Response

We thank the reviewer for highlighting that the colormap and color encodings in the figures should be stated explicitly. We revised the captions and associated text to clarify how the color maps and color overlays should be interpreted.

For Fig. 5, we clarified that the latent embedding density visualization uses a Viridis colormap, where brighter yellow corresponds to the highest density of vesicle instances in the latent embedding space. We also clarified that the clustering visualization uses a diverging blue-to-red density map to illustrate the separation of clusters identified by the HDBSCAN algorithm.

For Fig. 6, we revised the caption to explicitly define the color assignments used in the 3D renderings, specifying the target neuron, adjacent neuron fragments, bounding box, and expanded mask used to define the "near another neuron" region.

Additionally, we clarified the colormap encodings used in Fig. 7, where vesicle subtype and density are visualized using color mappings in the PyVista, Plotly, and three.js visualizations.

Where addressed in manuscript

Fig. 5 caption

> "Embedding distribution heatmap utilizing a Viridis color map, where yellow indicates the highest density of vesicle instances."

"HDBSCAN clustering of vesicle types overlaid on a density map using a diverging blue-to-red color map to visualize cluster separation."

Fig. 6 caption

"(A) 3D rendering of a neuron (green) within its minimal bounding box (yellow).
(B) The same neuron (green) with adjacent neuron fragments (orange) that fall within the bounding box.
(C) Expanded adjacent neuron masks (blue) used to define the spatial region considered 'near another neuron' within the target neuron volume."

Fig. 7 caption

"PyVista supports high-fidelity local mesh rendering with customizable colormaps, here representing vesicle subtypes."

"Three.js provides scalable full-dataset visualization with flexible metadata mapping, demonstrated here with a density-based colormap."

Comment 3

The evaluation metrics should be clarified, for example, the definition of RAND, precision and recall, and how these metrics assess the performance of an algorithm? The larger the bettor or in the other way around. F1 score should be included for a balanced evaluation.

Response

We thank the reviewer for this suggestion. In the revised manuscript, we clarified the definitions and interpretation of the evaluation metrics used to assess segmentation performance and included the F1 score as a balanced metric.

Specifically, we added explanations of the Foreground-Adjusted Rand Index (RAND), Precision, Recall, and F1 score, and clarified whether higher or lower values indicate improved performance. The text now explicitly explains that the RAND index measures the topological similarity between predicted and ground-truth labels (with lower values indicating better

agreement), while precision and recall quantify over-segmentation and under-segmentation tendencies, respectively. The F1 score was added to provide a balanced summary metric representing the harmonic mean of precision and recall.

These clarifications ensure that readers can properly interpret the segmentation evaluation results reported in Table 1.

Where addressed in manuscript

Large Vesicle Instance Segmentation: Instance segmentation

Lines: **116-121**

Added explanation of evaluation metrics:

> “To assess model performance, we utilize the Foreground-Adjusted Rand Index (RAND), Precision, Recall, and the F1 score. The RAND index (lower is better) measures topological similarity between predicted and ground-truth labels. Precision and Recall (higher is better) quantify over-segmentation and under-segmentation, respectively. Finally, we include the F1 score as a balanced metric to evaluate the harmonic mean of precision and recall across the dataset.”

Table 1

Added metric column:

> “F1 Score”

and clarified in caption:

> “Evaluation of model performance across checkpoints using RAND, precision, recall, and F1 scores.”

Comment 4

> The authors may provide more details of the training, validation and testing datasets, for example, how many vesicles of each category are contained in each subset for each training step?

Response

We thank the reviewer for this suggestion. In the revised manuscript, we clarified how annotated volumes were partitioned into training, validation, and testing subsets and described the iterative annotation–training workflow used to expand the training dataset across cycles.

Specifically, annotated ground-truth volumes were partitioned using an approximate 80/10/10 split into training, validation, and testing subsets. During model development, the dataset was expanded iteratively by identifying failure cases in model predictions, manually correcting these regions, and incorporating the corrected subvolumes into subsequent training cycles.

Because the training dataset was constructed through this iterative annotation process, the exact number of vesicles belonging to each class in each partition was not consistently recorded during early development stages. Instead, dataset scale is characterized in terms of the number of annotated volumes and voxel counts, which more directly reflect the spatial context used to train the segmentation model.

Where addressed in manuscript

Large Vesicle Instance Segmentation: Iterative training and prediction

Lines: **80-82**

Dataset partitioning:

> "Firstly, we annotated multiple ground truth volumes and partitioned them into training, validation, and testing subsets using an approximate 80/10/10 split."

Comment 5

> The results shown in Table 1 seems disagree to the content of figure 3. For example, in the caption of figure 3(B), it shows the results obtained from the experiments of 100,000 training epochs.

Response

We thank the reviewer for identifying this inconsistency. In the original manuscript, the training checkpoints shown in **Fig. 3** were incorrectly labeled and did not consistently correspond to the checkpoints reported in **Table 1**.

In the revised manuscript, we corrected the **panel labels and caption of Fig. 3** so that the segmentation results shown in panels **B–D** correspond directly to the training checkpoints reported in Table 1. Specifically, the panels now correspond to the model predictions after **100,000**, **300,000**, and **1,000,000** training iterations, respectively.

These corrections ensure that the example segmentation outputs shown in Fig. 3 are consistent with the training checkpoints reported in Table 1.

Where addressed in manuscript

Fig. 3 caption

Corrected panel descriptions:

> "(B) Prediction after 100,000 iterations… (C) Result after 300,000 iterations… (D) Result after 1,000,000 iterations."

Table 1

Training checkpoints now reported as:

> "Training Iterations: 100,000; 300,000; 1,000,000."

Comment 6

> Please carefully check the definitions of "training iterations (steps)" and "training epochs". Regarding "one epoch", we usually mean using all training data to iteratively train the model for one time. 1,000,000 epochs seems an extremely long training process.

Response

We thank the reviewer for raising this point. The values reported in Table 1 represent training iterations (gradient update steps) rather than epochs over the full dataset. Thus, the reported

checkpoints of 100,000; 300,000; and 1,000,000 correspond to optimization steps performed during model training.

To clarify this distinction, we added explanatory text describing the difference between training cycles and training iterations. In our workflow, a *training cycle* refers to one round of annotation, model training, prediction, and human correction used to iteratively improve the model, while *training iterations* refer to the individual gradient update steps performed during model optimization.

We also clarified the computational scale of the training process in the Results section.

Where addressed in manuscript

Large Vesicle Instance Segmentation: Iterative training and prediction

Added clarification:

> "Here, we use the term training cycle to denote one full round of annotation, model training, prediction, and human correction. In contrast, the training iterations reported in Table 1 refer to individual gradient update steps performed during model optimization."

Results section

> "In our experiments, model training required approximately 10 hours using four NVIDIA A10 GPUs."

Comment 7

> The title of figure 4B should be DCV not DV. And the items (A) (B) (C) are also missing in this figure.

Response

We thank the reviewer for pointing out these labeling errors. In the revised manuscript, we corrected the vesicle type label in Fig. 4B from "DV" to "DCV" (Dense Core Vesicle) to ensure consistency with the terminology used throughout the manuscript.

Additionally, we updated Fig. 4 to explicitly include panel labels (A), (B), and (C) corresponding to the three vesicle categories displayed in the proofreading interface. The caption has been revised accordingly to clearly reference each vesicle type shown in the figure.

Where addressed in manuscript

Fig. 4 caption

> “The interface enables human-in-the-loop verification of automated vesicle classifications. For each candidate (A) Clear vesicle (CV), (B) Dense core vesicle (DCV), or (C) Dense core vesicle with a halo (DCVH), the interface presents five consecutive Z-slices (Slices 0–4) overlaid with the contour of the 3D U-Net prediction in red…”
